# Non-genetic impact factors on chronological lifespan and stress resistance of baker’s yeast

**DOI:** 10.15698/mic2016.06.504

**Published:** 2016-04-12

**Authors:** Michael Sauer, Diethard Mattanovich

**Affiliations:** 1 Department of Biotechnology, BOKU – VIBT, University of Natural Resources and Life Sciences, Vienna, Muthgasse 18, 1190 Vienna, Austria.; 2 Austrian Centre of Industrial Biotechnology, Muthgasse 11, 1190 Vienna, Austria.

**Keywords:** Yeast, chronological lifespan, aging, stationary culture, oxygen

## Abstract

Survival under nutrient limitation is an essential feature of microbial cells, and it is defined by the chronological lifespan. We summarize recent findings, illustrating how crucial the choice of the experimental setup is for the interpretation of data in this field. Especially the impact of oxygen supply differs depending on the culture type, highlighting the differences of alternatives like the retentostat to classical batch cultures. Finally the importance of culture conditions on cell aging and survival in biotechnological processes is highlighted.

## INTRODUCTION

**Figure 1:**
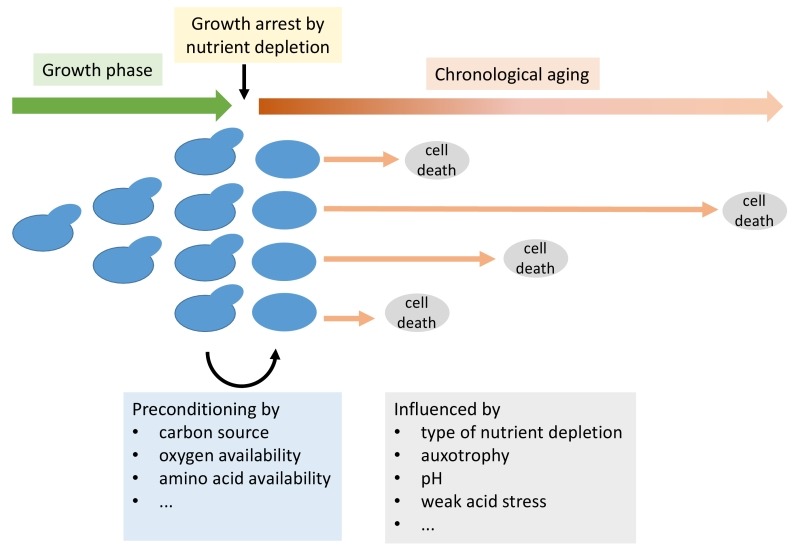
FIGURE 1: Schematic representation of growth and chronological aging of yeast cells with an indication of factors influencing the chronological lifespan.

 Chronological aging defines the survival of cells in stationary phase after nutrients become limiting and cells no longer divide 1 (Figure 1). Cells in this phase of non-division are interesting because they are known to be more stress resistant than fast growing cells. However, while the cells are more resistant to sudden detrimental changes in their environment, such as oxidative stress or heat shock, they nevertheless die after a while – also under ideal conditions - due to the aforementioned chronological aging. The measure for this is the chronological lifespan (CLS). These cells are furthermore interesting as this state of non-division is generally important for biology. While the natural habitat of many yeast species remains a question for debate, it can be speculated that the natural state of yeasts is non-dividing under nutrient scarcity for long periods of time, wherever they are found. Furthermore, many industrial applications involving baker’s yeast, such as wine making or ethanol production, take place at least partially under conditions of no-growth.

The ability to survive a period of famine and reproduce again upon re-feeding is influenced by a variety of internal and external parameters. While some light has been shed on the genetic program of aging, less is known about the environmental impact on chronological aging in baker’s yeast, particularly because the experiments were often not designed to obtain this knowledge.

## FACTORS INFLUENCING CHRONOLOGICAL LIFESPAN

Caloric restriction, that is nutrient limitation without malnutrition, is up to now the most prominent non-genetic intervention known to prolong the CLS 1. In fact, the CLS of yeast cells exposed to glucose under experimental conditions, which do not allow cell growth (such as lack of an essential amino acid) is dramatically reduced, when compared to cells which are arrested due to the absence of a carbon source. However, not all carbon sources have the same effect. Exposure of yeast cells to glycerol under non-growth conditions has been shown to have no detrimental effect on CLS 2. An explanation for this behavior has been suggested by Burtner *et al.*, who could show that the one major cause for CLS analyzed under standard conditions, is the presence of acetic acid at low pH 3. Experiments to determine the CLS of yeast cells are usually done in defined, unbuffered medium. Baker’s yeast growing on glucose accumulates organic acids, among them acetic acid, thereby lowering the pH of the medium significantly (to 3 or even less). However, it was shown that the presence of acetic acid at low pH kills the cells rapidly. Buffering the pH or suspending the starving cells in water instead of spent medium significantly increases the CLS. Coming back to the carbon source, it becomes clear why growth on glycerol has a beneficial effect on CLS, as no acetic acid is produced under these conditions. The connection of CLS to acetic acid at low pH also brings along the explanation why stress resistance and CLS are tightly interconnected. Many stress reactions overlap. For example, it has been shown that stress resistance against high osmolarity at least partially leads to tolerance against organic acids at low pH. It makes therefore sense, that preconditioning of baker’s yeast cells at high osmolarity leads to a significantly prolonged CLS under the standard conditions. However, the stress caused directly by acetic acid might not be the only metabolic cause for a reduced CLS, as the acetate metabolism proceeds via the central metabolite acetyl-CoA, which is in turn a signaling molecule influencing autophagy and thereby interfering with another mechanism, which is essential for cell survival under nutrient depletion 4. A further connection between stationary phase and stress resistance is the cell cycle control. It has been proposed that tight control of the cell cycle progression is fundamental to prolonging lifespan 5. At the same time nutrient scarcity has a direct impact on cell cycle control, with nutrient sensing pathways as master regulators – thereby closing the connection.

## WHAT HAPPENS TO CELLS UPON NUTRIENT DEPLETION?

The interesting question is, what exactly happens when cells enter a phase of nutrient scarcity. The obvious and visible response is the cessation of cell division. However, much more must happen in order to guarantee the survival of the culture as long as possible. Stationary-phase cells must actively respond to these environmental changes and must remain metabolically and biosynthetically active – at least at reduced levels 6. Endocytosis, autophagy, and respiration play a crucial role for the mobilization of intracellular reserves and energy provision for survival. In fact, it is relatively simple to find correlations of certain biochemical traits with aging and lifespan, however it is extremely difficult to obtain proof for causal relationships.

It is emerging that new technologies and new approaches need to be applied to understand the underlying processes. First of all, it has to be acknowledged that a yeast culture in stationary phase is not made of uniform cells, which are “stationary”. At least two populations can be identified – quiescent and non-quiescent cells, which behave entirely different. A proper description of such cultures must take this fact into account 7. Furthermore, choice of the strain and definition of the experimental set-up are crucial for obtaining relevant results. Natural baker’s yeast strains are diploid and prototroph, while the typical laboratory yeast strain is haploid and auxotroph. The genetic setup directly interferes with the nutrient sensing pathways, thereby compromising the results – depending on what should be the conclusion. It has been shown for example, that certain genetic factors have opposite effects on the CLS under laboratory and winemaking conditions, pointing in a direction that not the actual nutrient concentration might be important, but the ratio in which nutrients are supplemented 8. The influence of the different auxotrophies of laboratory yeast strains on CLS has not been sufficiently analyzed yet, although it is evident that such an influence exists 9. First of all, the involved nutrient sensing pathways react not only to carbon sources but also to nitrogen sources such as amino acids, thereby dictating a connection. Another aspect is that yeast cells will take up amino acids when available, and repress their respective biosynthetic pathways. This clearly leads to a reprogrammed metabolic network with all its implications. More directly, it has been shown, that the concentration and type of amino acids added to the growth medium are decisive for a toxic effect of ammonium (another typical ingredient of laboratory media) on CLS 10. Which amino acids are present, finally depends on the strain used, which in turn is more often dictated by laboratory habit rather than rational choice. On top of all, CLS analyses in unbuffered media always include weak acid stress – a fact that needs to be taken into account when interpreting the results.

However, not only conditions, but also methods need to be scrutinized. It has been shown only recently using live-imaging technologies that active actin remodeling is an essential factor influencing CLS 11. Active actin points into the direction that energy availability is a crucial factor for survival also for resting cells. The most efficient way of energy provision is respiration and it has been shown that mitochondrial fractionation is a sign of cellular degeneration leading to death in stationary phase 12. It appears therefore also plausible that oxygen availability is beneficial for survival. However, the mitochondria and more particularly reactive oxygen species produced by these respiring organelles have also a central (negative) role in the aging of cells.

## OXYGEN AND CHRONOLOGICAL LIFESPAN

It has been reported that enhancing respiration and mitochondrial gene expression promotes chronological longevity 13. Under conditions of high glucose availability, baker’s yeast produces energy primarily via fermentation to ethanol. Only when glucose availability is low, yeast switches to a respiratory metabolism. Thus, a metabolic shift from fermentation to respiration is expected under conditions of caloric restriction, pointing to a connection between caloric restriction and use of oxygen. It has also been shown that a certain pre-adaptation to respiratory growth promotes a longer CLS 14.

Bisschops *et al.* looked into more detail of the transition from the growth phase into the stationary phase in presence and absence of oxygen to understand why the chronological lifespan of cells grown in anaerobiosis is reduced 15. In fact, the data presented point into the direction that oxygen availability itself is not the driving force for better survival (and increased heat stress tolerance) of aerobically grown cells of baker’s yeast. The key-point seems to be kind of a caloric restriction of these cells, which grow diauxic first on glucose, then on ethanol. The growth rate on ethanol is significantly lower as compared to growth on glucose and the transcriptome is already remodeled during growth on ethanol into the direction of the stationary phase. This remodeling does not take place in anaerobic cells. They grow with high growth rate until the glucose is consumed – then they suddenly stop growing, since ethanol is a non-fermentable carbon source. This leaves the cells somehow unprepared when glucose is consumed. This result has direct impact on industrial processes relying on yeast survival in a late fermentation phase, such as winemaking or bioethanol production. We conclude that microaeration would allow for the required slow growth in this phase and lead to proper adaptation for the stationary phase.

Interestingly, Bisschops *et al.* show that cells kept in a retentostat cultivation under anaerobiosis exhibit a CLS comparable to cells grown in simple batch under aerobic conditions, proving that the preparation of the cells by gradually slowing down the growth rate is a key-factor and this is hardly influenced by the availability of oxygen per se. However, it shall be underlined that a retentostat culture looks at cells with a growth rate approaching zero, which is not exactly the same as a stationary culture of cells. Still some exogenous energy is fed, even if at extremely low uptake rate. This means that maintenance energy is provided from outside even when zero growth is achieved, which separates this state from the true stationary phase of a culture, illustrating again the importance of a clear definition of the experimental setup.

## CONCLUSION

Particularly for biotechnology, but also to understand the ecology of yeasts, their ageing behavior in response to environmental factors is of eminent importance. However, the techniques used and the experimental setup employed to study yeast aging have to be appropriately designed to get the desired results. So was life imaging inevitable to determine the role of actin – even if microscopy with fixed cells is the standard procedure. The retentostat is necessary to unveil the true potential of baker’s yeast to survive in anaerobiosis – even if batch cultures are standard for physiological analyses. Heterogeneity of cultures needs to be taken into account – even if microbiologists traditionally tend to describe cultures as a number of uniform cells. Finally, the choice of the strain and the precise definition of the experimental setup might have to be re-evaluated, when the aim of the study is fundamental biological understanding.
